# How tree roots respond to drought

**DOI:** 10.3389/fpls.2015.00547

**Published:** 2015-07-29

**Authors:** Ivano Brunner, Claude Herzog, Melissa A. Dawes, Matthias Arend, Christoph Sperisen

**Affiliations:** ^1^Forest Soils and Biogeochemistry, Swiss Federal Institute for Forest, Snow and Landscape Research WSLBirmensdorf, Switzerland; ^2^Swiss Federal Institute of Technology ZürichZürich, Switzerland; ^3^Forest Dynamics, Swiss Federal Institute for Forest, Snow and Landscape Research WSLBirmensdorf, Switzerland

**Keywords:** abscisic acid, avoidance, carbon sequestration, hydraulic signals, molecular responses, mycorrhizas, tolerance, tree root traits

## Abstract

The ongoing climate change is characterized by increased temperatures and altered precipitation patterns. In addition, there has been an increase in both the frequency and intensity of extreme climatic events such as drought. Episodes of drought induce a series of interconnected effects, all of which have the potential to alter the carbon balance of forest ecosystems profoundly at different scales of plant organization and ecosystem functioning. During recent years, considerable progress has been made in the understanding of how aboveground parts of trees respond to drought and how these responses affect carbon assimilation. In contrast, processes of belowground parts are relatively underrepresented in research on climate change. In this review, we describe current knowledge about responses of tree roots to drought. Tree roots are capable of responding to drought through a variety of strategies that enable them to avoid and tolerate stress. Responses include root biomass adjustments, anatomical alterations, and physiological acclimations. The molecular mechanisms underlying these responses are characterized to some extent, and involve stress signaling and the induction of numerous genes, leading to the activation of tolerance pathways. In addition, mycorrhizas seem to play important protective roles. The current knowledge compiled in this review supports the view that tree roots are well equipped to withstand drought situations and maintain morphological and physiological functions as long as possible. Further, the reviewed literature demonstrates the important role of tree roots in the functioning of forest ecosystems and highlights the need for more research in this emerging field.

## Introduction

The ongoing climate change is characterized by increased temperatures and altered precipitation patterns. In addition, the frequency, intensity and duration of extreme climatic events, such as droughts, floods, and storms, has increased in recent decades, and a continuation of this trend is predicted (IPCC, 2007, 2014; Cavin et al., 2013). These changes in environmental conditions are affecting terrestrial ecosystems worldwide and have led to a reduction in the global net primary production (Zhao and Running, 2010). Specifically, negative impacts on forest health associated with water limitation are major contributors to forest declines at a global level (Allen et al., 2010; Smith, 2011; Choat et al., 2012; Anderegg et al., 2013b).

Drought is a multidimensional environmental constraint that can provoke tree responses from the molecular to the forest stand level (Hamanishi and Campbell, 2011). Negative impacts of drought are observed in many aspects of forest health including seedling recruitment, productivity and mortality of larger/mature trees, susceptibility to pathogen or insect attack, and vulnerability to damage from fire (Zhao and Running, 2010; Reichstein et al., 2013). Consequently, there is considerable demand for an improved understanding of how forest trees respond to drought in order to develop strategies for the preservation of forest tree growth and survival in the face of this particular environmental threat (Hamanishi and Campbell, 2011).

Tree root systems are key components of forest ecosystems: they are responsible for water and nutrient uptake, provide physical stabilization, store nutrients and carbohydrates, and provide C and nutrients to the soil through the process of fine-root turnover (Brunner and Godbold, 2007). In addition, roots may act as sensors for water-deficit conditions and send signals to shoots above ground (Hamanishi and Campbell, 2011). Recent reviews and commentaries about drought in connection with forests or trees demonstrate that most existing research has focused on aboveground tree parts (e.g., McDowell et al., 2008; Hamanishi and Campbell, 2011; Ryan, 2011; Harfouche et al., 2014). In contrast, the responses of tree roots to drought and their role under drought conditions remain unclear and are only marginally mentioned or even neglected entirely in such reviews. Roots are generally less well analyzed than aboveground organs because they are difficult to observe, particularly *in situ*, and methods tend to be laborious, imprecise, and difficult to standardize across experiments.

In total, roots are estimated to make up 20–40% of the biomass of trees (Jackson et al., 1997). Compared to herbaceous plants, root systems of forest trees are complex because they contain roots that differ in morphology and size. Coarse roots (>2 mm in diameter) are responsible for anchoring trees to the soil and serve as organs to transport water from deeper soil horizons. Fine roots (<2 mm in diameter) are important for the uptake of water and nutrients. Fine roots are typically described by several different traits such as biomass, lifespan, specific root length (SRL), C/N ratio, and lignin content. Because fine roots turn over, they provide carbon and nutrients to the soil and, thus, play an important role in carbon sequestration and carbon cycling (Brunner and Godbold, 2007). There is increasing evidence that drought can influence the structure and growth of both coarse and fine tree roots (Kozlowski and Pallardy, 2002). In this review, we summarize and discuss the current understanding of how roots of forest trees respond to dry conditions. We first summarize growth, anatomical, physiological, biochemical, and molecular responses. We then elucidate the role of mycorrhizas in drought resistance, and finally we assess the effects of drought on root traits and the potential consequences for root decomposability, soil organic matter (SOM) formation and SOM persistency.

## Drought Avoidance and Drought Tolerance

**FIGURE 1:**
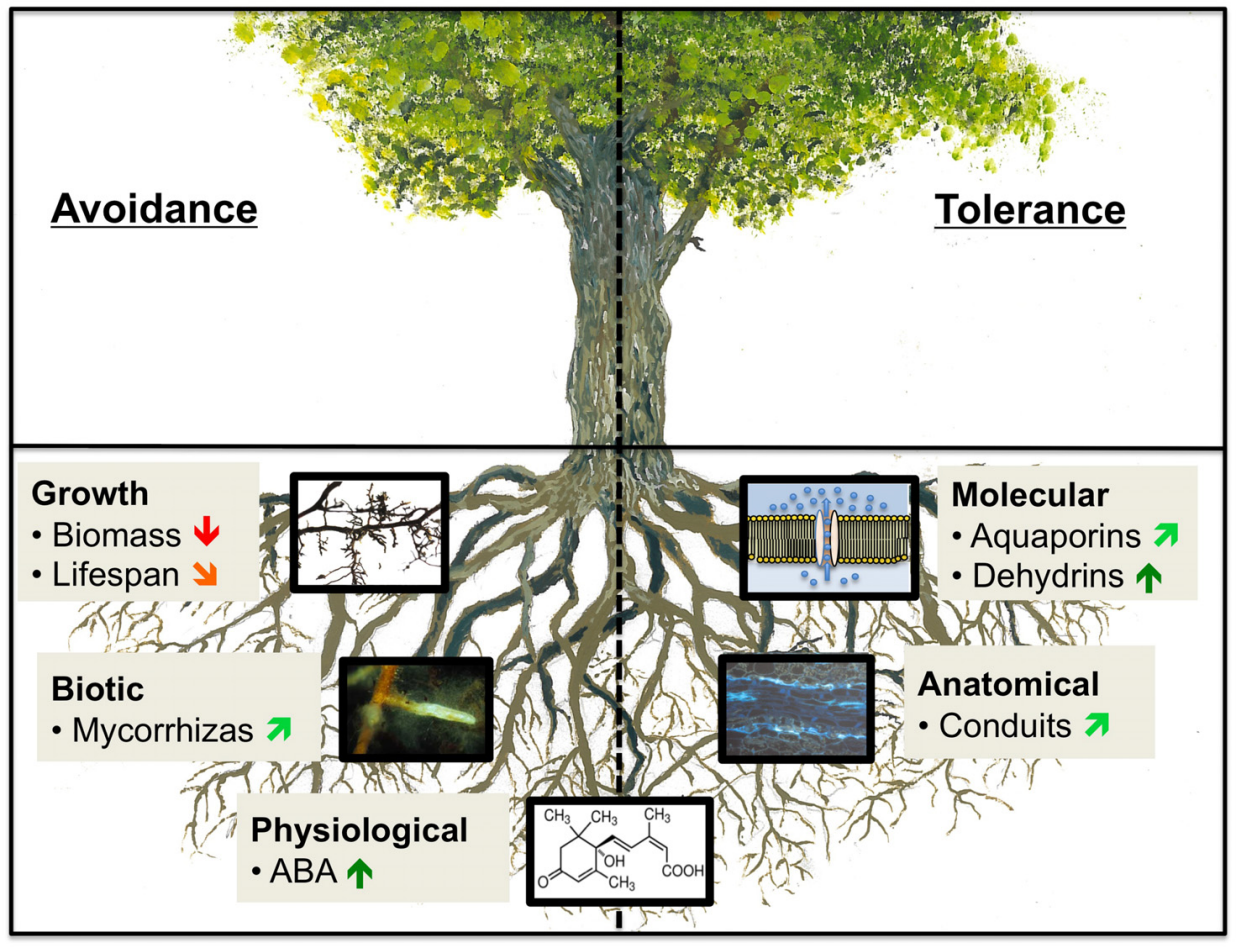
**Mechanisms of drought resistance and selected examples of tree root traits that respond to drought with avoidance or tolerance**. 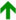
indicates a positive effect, 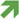
indicates a predominantly positive trend, 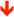
indicates a negative effect and 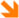
indicates a predominantly negative trend. Categories of tree root traits correspond to those given in Table 1. ABA, abscisic acid.

Evidence from physiological, biochemical, and molecular studies suggest that plants have evolved a variety of strategies to cope with drought. These strategies can be divided into those that enable plants to avoid low water potentials and those that enable them to tolerate dehydration (Levitt, 1972; Verslues et al., 2006; **Figure 1**). Strategies to avoid low water potentials rely on mechanisms that maintain the plant’s water status, such that the rates of water loss and water uptake remain balanced. Water loss can be limited by stomatal closure and over the longer term by restricting shoot growth, leading to an increased root-to-shoot ratio. Water uptake can be increased through enhanced root fine growth, formation of deep taproots, and accumulation of solutes to lower the water potential in the root tissue. When drought levels becomes too severe and drought avoidance mechanisms are no longer sufficient, plants respond by activating mechanisms that protect tissues against cellular damage, mainly through the action of protective solutes and proteins (Tuberosa, 2012; Claeys and Inzé, 2013). The physiological integrity of a plant is preserved as long as avoidance and/or tolerance mechanisms are adequate to avoid damage to cellular functions.

Unlike herbaceous plants, woody plants are characterized by extensive secondary growth, which itself can respond to drought conditions. For example, the diameter of the xylem conduits, responsible for the transport of water, and the thickness of their cell walls can be modified, resulting in increased resistance against cavitation in the vascular tissues (Kozlowski and Pallardy, 2002). Consequently, trees seem to have evolved mechanisms to cope with dehydration conditions that are distinct from those of herbaceous plants.

## Growth Responses

It is well documented that tree species adapted to dry climatic regimes generally have higher root-to-shoot ratios and deeper root systems than species that are more suited to mesic climatic conditions (Kozlowski and Pallardy, 2002; Hartmann, 2011). A meta-analysis of forest and woodland species from temperate and tropical regions showed a significant increase in the root-to-shoot ratio with a decrease in annual precipitation, from about 0.2 at 3000 mm^.^year^-1^ to about 0.4 at 500 mm^.^year^-1^ (Mokany et al., 2006). Similarly, in a survey of 62 tropical tree species, seedlings from dry forests were found to have a higher belowground biomass and deeper roots than seedlings from moist forests (Markesteijn and Poorter, 2009). Therefore, tree species adapted to dry conditions tend to invest more biomass into longer-lasting root organs, thus optimizing water uptake, while simultaneously minimizing water loss from transpiration. These patterns have contributed to the hypotheses that trees respond to water deficit by increasing root-to-shoot ratios and rooting depth (Mainiero and Kazda, 2006; Poorter et al., 2012). Field and laboratory manipulation experiments have indeed shown that many tree species respond to dry conditions with altered allocation to root and shoot growth. In *Populus euphratica*, for example, gradual water depletion led to a reduction of shoot growth, while root growth was maintained, particularly in the early phase of water depletion, leading to an increased root-to-shoot ratio (Bogeat-Triboulot et al., 2007). Results of a meta-analysis of a large number of experiments, including studies focused on trees, indicate that this growth response is strongly influenced by the severity of the stress (Poorter et al., 2012). Plants exposed to moderate water deficit generally show little change in their growth pattern, with only a small increase in the root biomass relative to the total biomass [root mass fraction (RMF)]. It seems that plants maintain their aboveground growth, and thus their competitiveness for aboveground resources, as long as possible under moderate drought. In contrast, plants exposed to severe drought, i.e., when the biomass is reduced by >50% of that of control plants, respond with a strong increase in RMF, largely due to a decrease in the growth of the stems (Poorter et al., 2012).

Variability in growth responses among studies may also be a consequence of genetic variation at both the species and population level. For example, research carried out on two poplar species revealed significant differences in the root-to-shoot ratio under drought treatment (Yin et al., 2004). Similarly, in a model ecosystem experiment including young oak trees, shoot growth responded more sensitively to drought than root growth, but this growth response differed between populations (Arend et al., 2011; Kuster et al., 2013).

Although the root-to-shoot ratio tends to increase under drought conditions, the biomass of fine roots in particular is often reduced as a consequence of reduced transpiration and respiration rates. This response has been shown repeatedly using field and laboratory experiments (e.g., Joslin et al., 2000; Konôpka et al., 2007; Rühr et al., 2009; Eldhuset et al., 2013; Herzog et al., 2014; Zang et al., 2014), across precipitation gradients (Leuschner et al., 2004; Meier and Leuschner, 2008a), and in meta-analyses of a number of studies (Cudlin et al., 2007; Yuan and Chen, 2010). Along with reduced fine root biomass, root length and root tip frequency typically decrease as well (e.g., Rühr et al., 2009; Eldhuset et al., 2013; Zang et al., 2014). However, other root traits, such as SRL, root tissue density (RTD), and root area index (RAI), often remain unchanged, as shown for oak (*Quercus* sp.) saplings exposed to drought (Arend et al., 2011) and in a semi-arid pine (*Pinus sylvestris*) forest that was irrigated (Herzog et al., 2014). A recent meta-analysis indicated that SRL seems to be especially resistant to drought, although SRL values varied greatly among the analyzed studies (Ostonen et al., 2007). On the other hand, a greenhouse experiment using 1-year-old seedlings of ten different tree and shrub species revealed that fine roots (diameter < 2 mm) had a reduced biomass under drought conditions, whereas very fine roots had an increased biomass (diameter < 0.5 mm; Olmo et al., 2014). In the same study, very fine roots had an increased SRL and RTD under drought but a smaller mean root diameter and a lower N concentration.

Whether a tree maintains old roots or sheds old roots and produces new ones in response to drought is, according to Eissenstat et al. (2000), determined by the benefit to cost ratio in terms of water uptake and carbon expenditure. Root shedding and the construction of new roots mean the investment of a considerable amount of energy in the process of root turnover. The benefit, however, is that young roots are able to take up water more efficiently than older ones, and thus, root shedding and regrowth represent a more suitable acclimation of plants to reduced water supply if the plant can afford this strategy. In a throughfall exclusion experiment using sub-canopy roofs, Gaul et al. (2008) concluded that fine root production was stimulated under mild drought (soil water matrix potential of -0.06 MPa), suggesting a compensation for enhanced mortality due to water shortage, whereas fine root mortality occurred without replacement under stronger drought conditions (-0.12 MPa). However, other factors, such as soil conditions and climate, may strongly influence the response of fine root systems to reduced soil water content (Gaul et al., 2008). For example, Persson et al. (1995) observed that fine roots of Norway spruce trees in Sweden shifted into deeper mineral soil horizons in response to experimentally induced drought, whereas Blanck et al. (1995) did not find such redistribution in a similar experiment in Germany with the same species. Finally, in a study with young oak trees, Kuster et al. (2013) found a redistribution of root biomass under drought conditions and a general reduction of the total root biomass.

Having a reduced root biomass under drought conditions does not necessarily imply that root production and root turnover rate are reduced as well. There are various methods and formulae to assess and calculate turnover rates of roots, and therefore results are often controversial (Gaul et al., 2009; Gaudinski et al., 2010; Lukac, 2012; Brunner et al., 2013; Ahrens et al., 2014). In their study of a *Picea abies* forest using rainfall exclusion treatments, Gaul et al. (2008) found a slightly higher turnover rate in the drought treatment compared to the control treatment, whereas Brunner et al. (2009) did not find any significant changes in turnover rate with an irrigation treatment in a *Pinus sylvestris* forest in a semi-arid area. On a global scale, root production and turnover rate were not correlated with mean annual precipitation but with mean annual temperature (Gill and Jackson, 2000; Finér et al., 2011). However, a meta-analysis by Yuan and Chen (2010) indicated a slightly significant correlation of tree root production and turnover rate with mean annual precipitation: lower water availability corresponded to a lower root turnover rate.

Analyses of root lifespan, the reciprocal value of turnover rate, indicate that lifespan tends to become shorter if water availability is hampered (Eissenstat et al., 2013). McCormack and Guo (2014) proposed a conceptual model indicating that root lifespan most likely depends on water availability. Adding water and alleviating drought should increase whole-plant productivity and increase root lifespan (Mainiero and Kazda, 2006; Meier and Leuschner, 2008b). However, additional water applied to an environment that already has adequate moisture may in fact reduce root lifespan (Leppälammi-Kujansuu et al., 2014), as a greater frequency of anoxic conditions increases root stress and pressures from external factors (McCormack and Guo, 2014). As suggested by Prieto et al. (2012), hydraulic redistribution may also play a role in mediating responses of fine roots to drought. In a study by Bauerle et al. (2008) using the woody shrub *Vitis vinifera*, the lifespan of fine roots growing in dry soil was reduced in the absence of hydraulic redistribution. However, when conditions allowed for a redistribution of water from wetter soil to dryer soil, the lifespan of roots in dry soil was maintained at the same level as roots under conditions without water stress (McCormack and Guo, 2014). In a review of root lifespan, Chen and Brassard (2013) found that fine root lifespan did not change with soil water addition when evaluated using pooled lifespan data but was marginally longer with water addition when evaluated based on the size of the treatment effect.

A compilation of the above-mentioned root traits of trees and the responses to drought is given in **Table 1**

**Table 1:** Effects of drought on tree root traits and potential consequences for root decomposability, soil organic matter (SOM) formation, and SOM persistency.

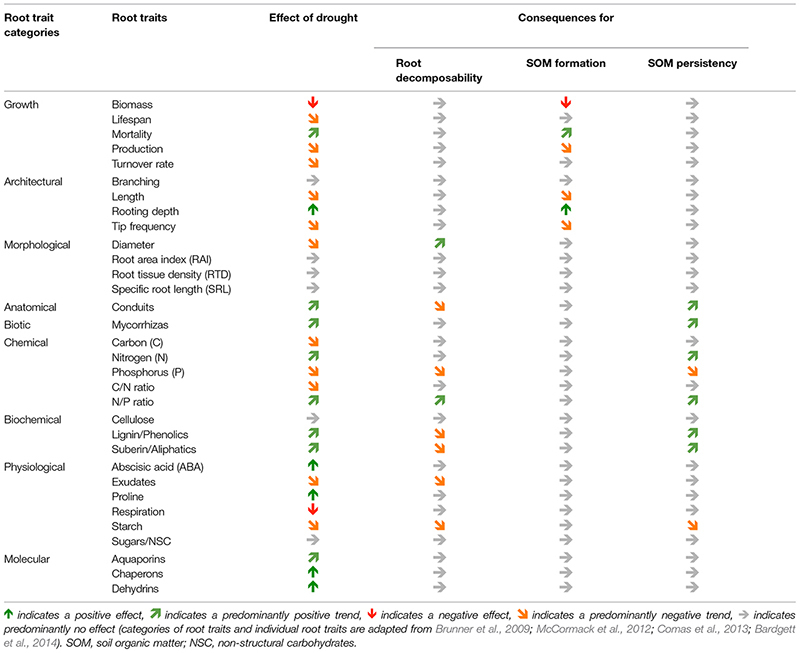

## Anatomical Responses

Water transport in trees is dependent on plant branching architecture, plant size, and plant developmental stage (Mencuccini, 2015). Compared to stems and branches, studies on the anatomical characteristics of the water transport system in roots are very limited. Xylem conduits are assumed to narrow from the roots to the stem and further to the branches and leaf petioles, in order to achieve an optimal structure for the functioning of the vascular elements (e.g., Tyree and Zimmermann, 2002). This assumption was confirmed in recent studies, where the xylem conduit diameters in roots and in aboveground parts of temperate, boreal, and tropical tree species were investigated (e.g., Lintunen and Kalliokoski, 2010; Schuldt et al., 2013). Conduit tapering is commonly believed to control water distribution. By having the lowest conductivities in the minor branches at the end of the flow path, a plant can control the distribution of water regardless of the transport distance. As the most distal organs belowground, fine roots can be sacrificed in response to drought, similar to leaf fall, as observed in various temperate and boreal forests (e.g., Gaul et al., 2008; Chenlemuge et al., 2013; Hertel et al., 2013). Kotowska et al. (2015) postulated a mechanism for fine root die off analogous to that of leaf abscission by defining a ‘hydraulic fuse,’ a concept evolved from Zimmermann’s segmentation hypothesis (Tyree and Zimmermann, 2002). At the root level, this ‘hydraulic segmentation’ might additionally protect the belowground system from a reverse water flow from the roots back to the dryer soil (Kotowska et al., 2015).

Due to secondary growth, trees have the ability to respond to low water conditions by modifying their vascular tissues (Kozlowski and Pallardy, 2002; Fonti and Jansen, 2012). At the level of individual conduits, there is a tight proportionality between conduit wall reinforcement and cavitation resistance (Hacke et al., 2001). Additionally, the formation of smaller vessels under drought has been observed in stems of various tree species, such as *Quercus* sp. and *Populus* sp. (Arend and Fromm, 2007; Fonti et al., 2013). It was further suggested that smaller vessels may be less susceptible to drought-induced xylem embolism and that they also contribute to the regulation of water flow under water-limiting growth conditions. To date, little is known about root anatomical responses to drought. Eldhuset et al. (2013) showed that tracheid diameters and hydraulic conductivity of young drought-stressed *Picea abies* were significantly lower and that tracheids were flatter in trees subjected to severe drought than in control trees, for both long and short roots. Both the reduction in tracheid size and the change to more ellipsoid tracheids might be responsible for the decrease in conductivity in the drought stressed roots (Eldhuset et al., 2013).

## The role of Abscisic Acid and Hydraulic Signals in the Drought Response

Evidence from physiological and genetic studies demonstrates that many physiological responses of plants to drought, avoidance as well as tolerance responses, are mediated by the plant hormone abscisic acid (ABA), although the underlying mechanisms are not fully understood yet (Munns and Cramer, 1996; Claeys and Inzé, 2013).

Abscisic acid is produced in roots as well as in leaves (De Smet and Zhang, 2013), and levels in both plant parts increase upon exposure to drought and are accompanied by major changes in gene expression and physiological responses, such as stomatal closure. ABA has been generally regarded as a hormone with different regulatory properties in growth and development. Under non-stressed conditions, low concentrations of ABA promote root meristem maintenance and root growth (Sharp et al., 2000), whereas under drought conditions enhanced ABA concentrations inhibit growth (Nakashima and Yamaguchi-Shinozaki, 2013). Based on this observation, ABA has been commonly considered a growth limiting stress hormone (Quarrie and Jones, 1977; Trewavas and Jones, 1991). However, the actual role of ABA as a growth-limiting hormone remains obscure, as contradictory findings suggest an opposite function (Sharp et al., 1994, 2000, 2004; Arend and Fromm, 2013). Genetic evidence that ABA plays a role in growth control comes from a study analyzing a transgenic poplar line that ectopically expresses the mutant *Arabidopsis abi1* gene (Arend et al., 2009). Expression of this gene resulted in an ABA-insensitive phenotype with enhanced shoot growth but retarded leaf and root development. This altered growth pattern supports the hypothesis that ABA acts independently from drought as a negative regulator of growth in shoots and as a positive regulator of growth in leaves and roots.

Abscisic acid is transported from roots to leaves, where it acts as a long-distance signal inducing the closure of stomata and triggers the expression of ABA biosynthesis genes and, thus, increases the ABA content in leaves (De Smet and Zhang, 2013). It is likely that both mechanisms are required to induce stomatal closure. However, recent studies question the role of root-born ABA in stomatal closure and instead propose electrical and hydraulic root signals (Christmann et al., 2007, 2013; Schachtman and Goodger, 2008). Electrical signals emanating from water-stressed roots, or from roots after re-irrigation as shown in young avocado plants (*Persea americana*), might be relayed independently from hydraulic signals (Grams et al., 2007; Gil et al., 2008). Root-derived hydraulic signals result in local water potential (Ψ) changes and, concomitantly, in turgor changes that can be compensated with solute adjustments (Christmann et al., 2007). The changes in Ψ are relayed into the inner stele of the roots and increase the tension on the water in the xylem vessels for rapid long-distance signaling. This hydraulic signal is transmitted along the root axis and perceived by parenchyma cells in the shoots, which are sites of ABA biosynthesis (Endo et al., 2008; Christmann et al., 2013). In the shoots, the hydraulic signal promotes the closure of stomata via a biochemical effect on guard cells and via an indirect hydraulic effect, i.e., a decrease in water permeability within leaf vascular tissues (Pantin et al., 2013).

Water always follows a water potential gradient, and thus dehydration in roots can be avoided through a reduction in hydraulic conductivity and osmotic potential. In this context, ABA in roots also mediates an enhancement of the biosynthesis of osmolytes, such as the amino acid proline, and of protective proteins (Davies and Bacon, 2003). However, only a few studies have demonstrated distinctly enhanced proline concentrations in the roots of trees after a drought treatment (Sofo et al., 2004; Cocozza et al., 2010; Naser et al., 2010). In particular, proline plays a dual role as both osmolyte and osmoprotectant (‘osmotic adjustment’; Kozlowski and Pallardy, 2002; Claeys and Inzé, 2013). Root water uptake and distribution is controlled by water channel (aquaporin) activity (Almeida-Rodriguez et al., 2011). Under drought conditions, ABA increases aquaporin expression, which translates into increased hydraulic conductance (water uptake at the soil-root interface; Parent et al., 2009).

**FIGURE 2:**
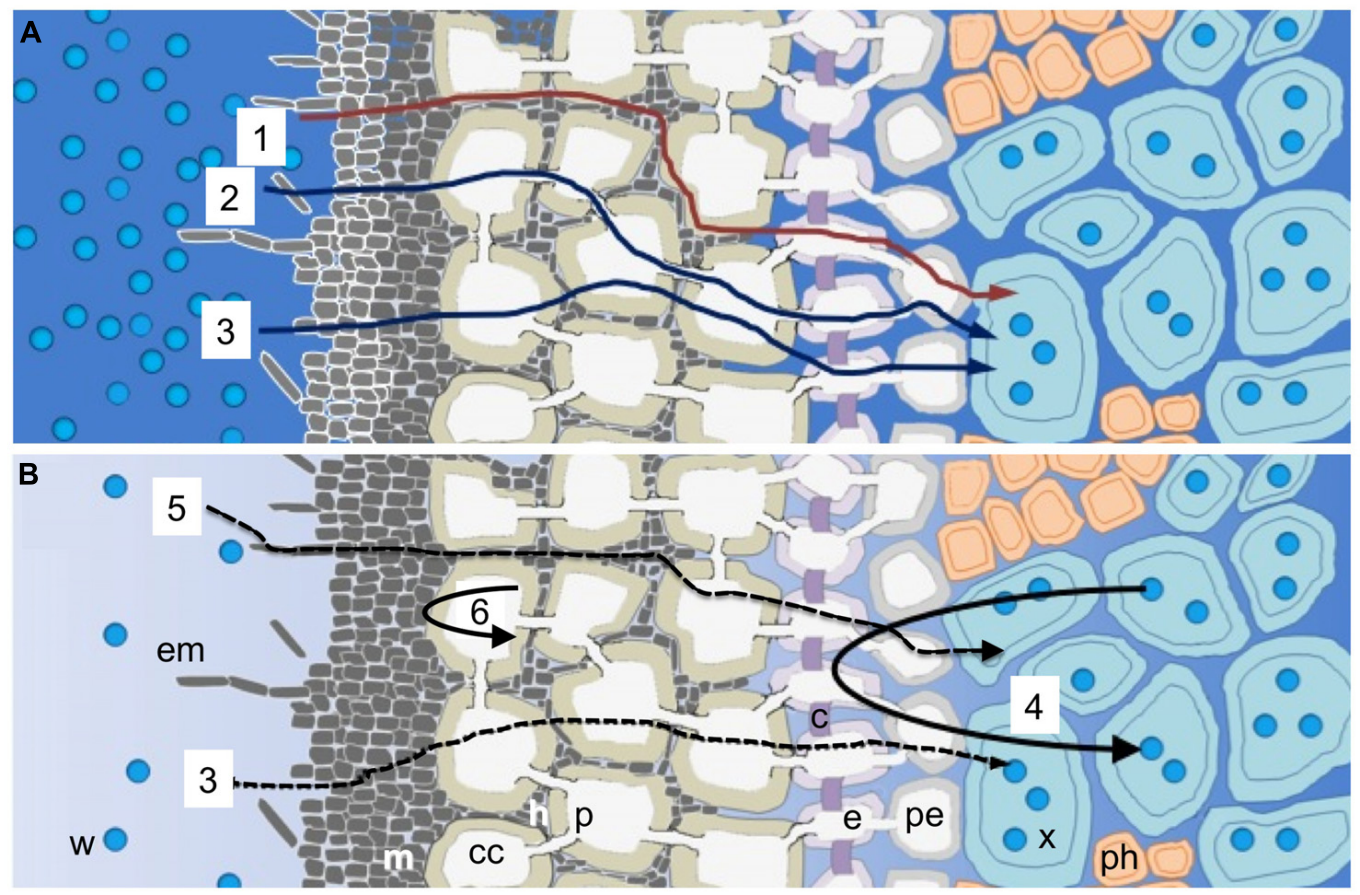
**Schematic diagram illustrating how water moves into mycorrhizal roots. (A)** Water movement under non-drought and **(B)** water movement under drought conditions. Non-drought conditions: water transport through the roots involves a combination of apoplastic (1), symplastic (2), and transcellular (3) pathways. Drought conditions: water flow is mainly transcellular (3), which causes a high hydraulic resistance as water passes across many membranes via aquaporins. Aquaporins then may act as valves to reversibly increase the hydraulic conductivity, and the suberisation of roots minimizes water loss to the dry soil (4) (according to Steudle, 2000). External mycelium of the fungal mantle facilitate water uptake (5), and melanised fungal hyphae prevent cortical cells from desiccation (6) (according to Lehto and Zwiazek, 2011; Fernandez and Koide, 2013). c, Casparian strip; cc, cortical cells; e, endodermis; em, external mycelium; h, Hartig net; m, fungal mantle; p, plasmodesma; pe, pericycle; ph, phloem; w, water molecules; x, xylem.

Water transport through the roots involves a combination of three different pathways: the apoplastic path (around the protoplasts), the symplastic path (through the plasmodesmata), and the transcellular path (across the cell membranes) (Steudle and Peterson, 1998; **Figure 2**). The transcellular water movement is tightly controlled by the amount and activity of water channels (aquaporins) in the cellular membranes (Chaumont and Tyerman, 2014). There is a rapid exchange of water between parallel radial pathways because, in contrast to solutes such as nutrient ions, water permeates cell membranes readily. The apoplastic barriers consist of the Casparian bands and the suberin lamellae in the exo- and endodermis of roots. By switching the apoplastic path on or off, water uptake is regulated according to demands from the shoots (Steudle, 2000). At high rates of transpiration, the apoplastic pathway is partially used and the hydraulic resistance of roots is low and evenly distributed across the root cylinder, allowing for rapid uptake of water. On the contrary, at low rates of transpiration such as during drought conditions, the apoplastic pathway is used less. Instead, water flow is mainly transcellular, which causes a high hydraulic resistance as water passes across many membranes via aquaporins in its passage across the root cylinder (Steudle, 2000). During conditions of water deficit, the suberisation of roots minimizes water loss to the dry soil. Aquaporins then may act as valves to reversibly increase the hydraulic conductivity and allow for water uptake under drought conditions. It has been shown that aquaporins can be activated by phosphorylation, which, in turn, is affected by factors such as Ψ, turgor, or Ca^2+^ concentration in the apoplast (Steudle, 2000).

## Non-Structural Carbohydrates, Carbon and Nitrogen

Important components of the C budget are non-structural carbohydrates (NSC) such as starch and sugars. Thus, the amount of NSC may reflect the drought status of trees, although such compounds are constantly consumed by fine-root production, respiratory metabolism, and osmotic adjustments, eventually leading to a decline in NSC concentrations in later stages of drought (Gaul et al., 2008; McDowell, 2011). A recent study of beech saplings (*Fagus sylvatica*) did not show such a relationship between drought and fine-root starch and NSC concentrations except fructose, although photosynthesis and rhizosphere respiration decreased with increasing drought (Zang et al., 2014). This increased concentration of fructose in the fine roots could be interpreted as a response to soil drought, as this sugar lowers the osmotic potential in the plant as a precursor to enhanced water uptake. Regier et al. (2009) applied drought to two contrasting *Populus nigra* clones and observed two different strategies: the drought-adapted ‘Poli’ clone had significantly more starch but significantly less sucrose, glucose, and fructose in the roots in the drought treatment, whereas the drought-sensitive ‘58-861’ clone had significantly less starch in the roots in the drought treatment, with the sugars remaining unchanged. It seems that the well-adapted clone from the dry site was not strongly stressed by water shortage, whereas photosynthesis was inhibited by drought in the less-adapted clone from the wet site, causing a reduction of carbohydrate allocation to the roots and inducing degradation of starch to maintain root respiration (Regier et al., 2009). In a study by Galvez et al. (2011) using seedlings of aspen (*Populus tremuloides*), it appeared that they were adapted to drought, because they were able to switch from growth to root reserves storage. The drought stressed seedlings showed a doubling of sugar and starch content in the roots, and roots of these seedlings contained more starch relative to sugar than those in the controls.

Contrasting results were shown in a drought experiment using two different *Nothofagus* species (*N. dombeyi*, *N. nitida*). Whereas the concentrations in the roots of *N. dombeyi* remained unchanged, *N. nitida* roots showed reduced starch and total soluble sugar (TSS) concentrations under drought conditions (Piper, 2011). An application of a lethal drought treatment to young *Picea abies* trees resulted in a significant decrease in starch and other sugars (Hartmann et al., 2013a), whereas plants that were watered again after a drought period (relative extractable soil water content below 3%) did not experience a significant change in starch and other sugar concentrations in the roots (Hartmann et al., 2013b). Mitchell et al. (2013) found a similar result when applying a lethal drought treatment to two eucalypt (*Eucalyptus globulus, E. smithii*) and one pine (*Pinus radiata*) species. Starch concentrations decreased significantly in the roots of all three tree species under drought, whereas the soluble sugar (SS) concentration decreased in the eucalypt roots but increased in the pine roots. All these results show that it is difficult to make general statements about NSC responses to drought.

Carbon (C) and nitrogen (N) concentrations in fine roots have been proposed to be associated with root functioning and are therefore often analyzed simultaneously with other root traits. In the review of Sardans et al. (2012) about stoichiometric features, the authors reported an altered C/N ratio in roots under drought in only one tree species. *Quercus ilex*, a mediterranean tree species growing in semi-arid areas, showed a drought-induced decrease in C/N ratio but an increase in N/P and C/P ratios, due to reduced C and P concentrations as well as increased N concentrations (Sardans et al., 2008). Similarly, in a more recent study by Herzog et al. (2014), irrigation of a drought suffering pine forest decreased the N concentration in fine roots; however, the C/N ratio and P concentration were not affected.

## Suberin and Lignin Formation in Roots Under Drought Conditions

Suberin is a complex and poorly characterized biopolymer of root cell walls comprised of both aliphatic (fatty acid derivative) and aromatic (phenylproponoid derivative) domains. It is an important component of endo- and exodermal cells, as well as cork cells of the periderm in woody plants (Soler et al., 2007; Ranathunge et al., 2011). The suberisation of roots minimizes water losses to the soil, in particular during conditions with a water deficit (Steudle, 2000). In addition, drought can induce the formation of suberin, as many different abiotic and biotic stresses can induce changes in cell wall composition (Moura et al., 2010). For example, it has been shown that the total concentration of aliphatic monomers in *Quercus ilex* roots from a dry environment is much higher than that in roots of the same species from a moist environment (Andreetta et al., 2013). Further, in roots of the grapevine *Vitis vinifera*, elevated suberin deposition was observed in drought-stressed plants (Vandeleur et al., 2009). Increased root suberin content was found to reduce daytime transpiration rates and increase water-use efficiency during the vegetative period (Baxter et al., 2009).

Lignin is a major component of the vascular plant cell wall, providing the mechanical support that allowed upright plants to adapt to a terrestrial habitat (Cabane et al., 2012). Drought apparently can result in wall tightening and thickening, as observed in the tracheids of drought-stressed Norway spruce roots (Eldhuset et al., 2013). Tightening appears to be caused by a number of mechanisms, including lignification of wall polymers (Moore et al., 2008). An increased amount of lignin improves the mechanical strength of cell walls in a dry environment, and cell wall lignification helps to minimize water loss and cell dehydration (Cabane et al., 2012).

## Molecular Responses

Recent transcriptome analyses in roots of *Populus* sp. and *Pinus* sp. underpin several of the metabolic changes identified in physiological and biochemical studies (Wilkins et al., 2009; Cohen et al., 2010; Lorenz et al., 2011; Perdiguero et al., 2012). Comparative analyses of different genotypes led to the identification of several major gene clusters and regulators that are important in the response of roots to drought. Of the interrogated genes, 8% (5331 transcripts) were differentially expressed in poplar and 9% (2445 transcripts) in pine (Cohen et al., 2010; Lorenz et al., 2011). In both species, genes involved in ABA biosynthesis and signaling were differentially expressed. In particular, the 9-cis-epoxycarotenoid dioxygenase (*NCED*) gene, catalyzing the first dedicated step in the biosynthesis of ABA, was induced in both species, indicating that root ABA levels increased in response to drought. In pine, numerous transcription factors known to play a role in drought and abiotic stress via ABA-dependent and ABA-independent pathways were differentially expressed. Genes encoding putative DREB1, bZIP, AP2/ERF, MYB, NAC, and WRKY transcription factors were all up-regulated, but none of them was identified as a central node. Nevertheless, one of *WRKY* genes was induced both during drought stress and in the recovery phase and was suggested to play a key role in regulating drought responses. In poplar, several of the *WRKY* genes identified were up-regulated. Consistent with the results of biochemical studies, the transcriptome analyses of both species identified several genes involved in the biosynthesis of osmolytes. In poplar, genes encoding sucrose synthase, galactinol synthase, and raffinose synthase were all up-regulated. Similarly, genes encoding galactinol and raffinose synthases were induced in pine. In addition, several genes of pine with putative functions in the biosynthesis of proline were induced.

A large gene family commonly identified in drought response studies is the late embryogenesis abundant (LEA) protein family, which includes dehydrins. Their precise functions are not known, but they are believed to be involved in a number of protective processes, including as hydration buffers, antioxidants and both enzyme and membrane stabilizers (Caruso et al., 2002). Dehydrins accumulate in root tissues in response to cellular dehydration caused by osmotic stress, and it is believed that they bind water in their random coil conformation and protect cellular structures from dehydration stress. The transcriptome analyses of both poplars and pines identified several members of this family to be induced. Similarly, several genes encoding chaperones/heat shock proteins (HSPs) and enzymes involved in scavenging reactive oxygen species (ROS) are up-regulated (Gosh and Xu, 2014).

Additional genes that were differentially expressed in the response of roots to drought included aquaporins. Aquaporins are a family of channel proteins that are found in cellular membranes and are responsible for water flux and thus play key roles in maintaining the water balance. The protein family can be grouped into five subfamilies, with the plasma membrane intrinsic proteins (PIPs) and the tonoplast intrinsic proteins (TIPs) representing the largest subfamilies (Gupta and Sankararamakrishnan, 2009). In poplar (*Populus trichocarpa, Populus balsamifera, Populus simonii x balsamifera*), a total of 56 aquaporins have been identified (Gupta and Sankararamakrishnan, 2009; Almeida-Rodriguez et al., 2010). Studies of the expression levels of aquaporins in roots using quantitative RT-PCR showed that an increase in root hydraulic conductance corresponded to increased transcript abundance of 15 aquaporins out of a total of 33 genes. A greater than twofold increase in expression level was found for two *PIP*s, two *TIP*s, and one *NIP*, whereas 2 *PIP*s showed a greater than twofold decrease in expression level (Almeida-Rodriguez et al., 2011). Root water flow and aquaporin expression patterns in hybrid poplars experiencing a sudden drop in humidity were shown to be tightly connected (Laur and Hacke, 2013). The rapid increase in root water flow corresponded to a 75% increase in the cumulative transcript copy numbers of all measured *PIP* genes, mainly due to a twofold increase in the transcript copy numbers of the *PIP1* genes, whereas no significant changes in the expression of *PIP2* genes occurred (Laur and Hacke, 2013). One gene, *PtPIP1;2*, which is consistently highly expressed in response to light (Almeida-Rodriguez et al., 2011), was also up-regulated in response to drought in the roots of *Populus nigra* (Cocozza et al., 2010). However, in the roots of the olive tree (*Olea europaea*), two *PIP* genes (*OePIP1;1, OePIP2;1*) were significantly down-regulated after water was withheld (Secchi et al., 2007). These results suggest that a single response of root *PIP* aquaporin expression and PIP protein abundance does not occur under drought conditions.

## The Role of Mycorrhizas in Drought Resistance

Ectomycorrhizal (ECM) symbioses may improve the water status of trees under drought conditions, through an increased absorbing surface, efficient conduction through mycelial strands, enhanced hydraulic conductivity at the soil-root interface, and hormonal and nutritional effects modifying stomatal regulation (Breda et al., 2006). According to Lehto and Zwiazek (2011), the clearest direct mechanism for increased water uptake is increased root extension and greater absorbing surface area through external mycelium, mainly by long-distance exploration mycorrhizal types. In a comparison of the root hydraulic conductivity of balsam poplar (*Populus balsamifera*) colonized by the ECM *Hebeloma crustuliniforme* with that of the same tree species colonized by the ectendomycorrhizal *Wilcoxina mikolae*, Siemens and Zwiazek (2008) observed a significant increase in root hydraulic conductivity by the presence of *H. crustuliniforme* but not *W. mikolae*.

Ectomycorrhizal fungi can additionally affect the cell-to-cell pathway through effects on plant aquaporin expression. Experiments on hybrid poplar (*Populus tremula x tremuloides*) seedlings inoculated with *Amanita muscaria* showed that for three of five investigated poplar *PIP* aquaporin genes, expression did not differ between ECM and non-ECM plants. However, expression of the two other aquaporin genes was more pronounced in roots of ECM plants, indicating that the root-fungus symbiosis may improve the water transport capacity under conditions of reduced water availability (Marjanović et al., 2005). However, ECM formation of the same poplar hybrid is also able to enhance the transcript abundances of aquaporin genes of the fungal symbiont. Transcript abundances of two genes of the ECM fungi *Laccaria bicolor* were enhanced more than twofold in the ECM formation, whereas gene expression of the other members of the aquaporin gene family were only minimally affected or, in one case, reduced by more than twofold (Dietz et al., 2011). In a study with jack pine (*Pinus banksiana*) seedlings inoculated with the ECM fungus *Suillus tomentosus*, a several-fold increase of the root hydraulic conductivity of ECM plants was observed compared to the non-ECM seedlings (Lee et al., 2010). This increase in water transport across the root cortex has been attributed to aquaporin-mediated transport, as measured by the indirect method of inhibiting the aquaporin activity with mercury. In another study using white spruce (*Picea glauca*) seedlings and the ECM fungi *Laccaria bicolor* wildtype (WT) or *L. bicolor* aquaporin over-expressing (OE) strain, OE plants had higher root hydraulic conductivity compared with WT plants and the increases were accompanied by higher expression of *P. glauca PIP* aquaporin expression in roots (Xu et al., 2015). Both WT and OE had increased shoot water potential, transpiration, net photosynthetic rate, root hydraulic conductivity, and root cortical cell hydraulic conductivity in comparison to non-ECM plants. These results lead to the conclusion that the contribution of *L. bicolor* hyphae to root water transport in *P. glauca* involves increased apoplastic water transport in the root intercellular spaces, which may lead to increased hydration at the fungal-root interface and, consequently, impact aquaporin expression and cell-to-cell water transport in ECM roots (Xu et al., 2015). As in the case of the basidiomycete *L. bicolor*, elevated aquaporin gene expression at the plant/fungus interface was also recently observed in hyphae of the ascomycete *Tuber melanosporum* (Hacquard et al., 2013). However, some studies have not shown positive effects of ECM fungi on aquaporin gene expression. A recent study using *Helianthemum almeriense* and its fungal symbiont *Terfezia claveryi* was conducted to investigate the expression patterns of five aquaporin genes from the plant and one from its fungal symbiont (Navarro-Ródenas et al., 2013). Results of this experiment indicated that the plant aquaporin genes were not enhanced in the roots by the ECM status and were even significantly reduced in one case. Further, Ψ of leaves of the ECM plants was not affected compared to non-ECM plants (Navarro-Ródenas et al., 2013).

The best-known indirect mechanism for ECM effects on water relations is probably improved nutrient status of the host due to facilitation of nutrient acquisition during drought. Other mechanisms include altered carbohydrate assimilation via stomatal function, possibly mediated by changes in growth regulator balance, increased sink strength in ECM roots, antioxidant metabolism, and changes in osmotic adjustment. None of these possibilities has been sufficiently explored. The ECM structure may also reduce water movement through improved fine root architecture (fungal mantle), cell wall hydrophobicity, or the larger number of membranes that water must cross on the way from the soil to the xylem (Lehto and Zwiazek, 2011). In addition, hydraulic redistribution can support nutrient uptake during prolonged dry periods. Soil water uptake displays a gradual downward shift as the soil dries, and a small fraction of total fine roots/ECM growing deeper in the soil ensures the overnight recovery of the soil-to-tree water potential equilibrium and supports a fraction of tree transpiration during periods of stomatal closure (Breda et al., 2006).

In a recent review, Mohan et al. (2014) concluded that the main effect of ECM associations is to reduce plant stress under drought conditions. They also indicated that the effects of the arbuscular mycorrhizal (AM) symbioses are similar to those reported for the ECM symbioses. Impacts of drought on AM and ECM abundance were mixed, with a slight majority of the studies finding decreased and a slight minority observing increased AM and ECM abundance with diminished soil moisture. Over half of the studies examining drought impacts on AM and ECM activity found no change in the rate of colonization, with the remainder nearly split equally between increased and reduced abundance. In most of the studies, AM and ECM increased plant productivity under drought conditions compared to non-AM/ECM plants. Similarly, while drought directly diminished rates of biogeochemical cycling in most of the studies, in two thirds of these studies biogeochemical rates were higher under drought conditions when plants were inoculated with AM and ECM compared with non-AM/ECM plants. In their meta-analysis on ECM, Cudlin et al. (2007) concluded that drought has a significant negative effect on ECM roots, although this effect was mainly due to the strong negative effect of drought on fine root biomass.

In a study of entire ECM communities, precipitation was found to have a significant effect on the ECM communities of oaks (*Quercus petrea, Q. robur*) throughout Europe, although pH and N-deposition were the main drivers (Suz et al., 2014). In contrast, Jarvis et al. (2013) found that precipitation and soil moisture had a strong influence on pine (*Pinus sylvestris*) ECM in Scotland, and they identified several taxa with variable abundances across the rainfall gradient. The resupinate species *Piloderma sphaerosporum* showed a strong decline in abundance with increasing rainfall, whereas the hypogeous fungus *Elaphomyces muricatus* showed a markedly greater abundance in forests experiencing large amounts of rainfall. Surveys in beech (*Fagus sylvatica*) stands in France demonstrated a larger sensitivity of *Lactarius* sp. to declining soil water potential compared to *Cenococcum geophilum* (Jany et al., 2003). The latter fungus infected free root apices and expanded, while the other ECM declined due to soil water shortage. *C. geophilum* is often mentioned as a particularly drought-tolerant fungus, but this statement has little support from experimental research (Breda et al., 2006). Recent studies suggest that the production of melanin (a class of complex dark polymers), also found in the fungal cell walls of *C. geophilum*, might be a key functional trait in water stress tolerance (Fernandez and Koide, 2013). Melanized cell walls may help prevent water molecules from leaving the cells, thereby increasing the success of the symbioses in resisting desiccation. Herzog et al. (2013) indicated that, although increased temperature and drought negatively affected the relative abundance and enzyme activity of *C. geophilum*, this fungus was able to tolerate severe drought.

## Tree Root Decomposability and the Role of Roots for Organic Matter Formation and Persistency

Root responses to drought clearly affect both the amount and traits of living and dead roots in forest ecosystems and, consequently, can have a strong influence on C dynamics and C sequestration in this environment. Living roots grow into soil and explore the soil matrix, and they constantly deposit decaying border cells and mucilage (rhizodeposition), exude enzymes, organic acids, ions and protons, and respire CO_2_ to the surrounding soil (Jones et al., 2009). In doing so, roots initiate the weathering of parental rock material and the cycling of nutrients and trace elements (Hinsinger et al., 2009). In addition to contributing to the weathering of rocks and nutrient cycling, living roots have a stimulating effect on the microorganisms that live in the surrounding soil known as the ‘rhizosphere priming’ effect (Kuzyakov, 2002, 2010). Root deposits and exudates fuel microorganisms with easily accessible carbohydrates, which in turn results in the exudation of microbial organic molecules and ions and ultimately leads to the decomposition and transformation of the surrounding organic matter.

Water deficit caused by drought could slow down root growth and reduce the amount of rhizodeposition and exudation. Drought might also alter the quality of roots by enhancing suberin and lignin contents, two key compounds affecting root decomposability, which could influence the transformation of root material into SOM (Von Lützow et al., 2006). Under the ongoing global change, with higher temperatures and altered precipitation patterns, it is likely that shifts in SOM composition may result in overall changes in SOM quality and turnover (Pisani et al., 2014). In particular, the accumulation of aliphatic root-derived compounds could be relevant because it gives SOM a hydrophobic protection (Dignac and Rumpel, 2013). Lignin, a second important compound, is mainly only degraded by a specific group of microorganisms, white-rot fungi and actinobacteria, which are able to secrete ligninolytic enzymes (Osono, 2007; Baldrian, 2008; Floudas et al., 2012). As a consequence, lignin content, and in particular the lignin/N ratio, is one of the driving forces in the decomposition of fine roots (Silver and Miya, 2001; Heim and Frey, 2004; Klotzbücher et al., 2011; Aulen et al., 2012; Talbot and Treseder, 2012; Talbot et al., 2012; Walela et al., 2014). Drought might affect the secretion and the activity of these extracellular ligninolytic enzymes, as the activity of such enzymes can vary considerably (Snajdr et al., 2011; Baldrian et al., 2013).

Alternatively, drought might shorten the lifespan of roots and accelerate root turnover (McCormack and Guo, 2014). After death, fine roots deliver a considerable amount of dead organic material to the decomposition process, and root-derived SOM plays a major role in C sequestration of forest soils (Rasse et al., 2005). However, under conditions of water deficit, dead roots are not decomposed completely and are instead transformed to a mor or moder type of humus. This can occur because the ‘rhizosphere priming’ effect is hampered (due to low rhizodeposition and exudation rates and due to low microbial activity), because the roots are more difficult to decompose (due to higher lignin and suberin content), or because key microorganisms such a lignin-degrading fungi are missing or inactive (Kuzyakov, 2010). In contrast, under conditions of sufficient water availability, dead roots are completely decomposed and transformed to a mull type of humus. As mentioned above, shifts in SOM composition might have long-term effects on SOM quality and turnover (Pisani et al., 2014).

As an intermittent disturbance of the water cycle, drought interacts with the C cycle differently than other ‘gradual’ changes in climate. During periods of drought, plants use species-specific strategies to respond physiologically and structurally in order to prevent excessive water loss. These responses have clear consequences for C uptake and release by plants. After a drought period, disturbances to soil moisture, organic matter, and nutrient content in the soil, and carbohydrate content in plants lead to longer-term effects on plant C (Van der Molen et al., 2011).

A compilation of root traits and consequences of drought on root decomposability, SOM formation, and SOM persistency is given in **Table 1**

## Feedbacks and Premature Mortality

**FIGURE 3:**
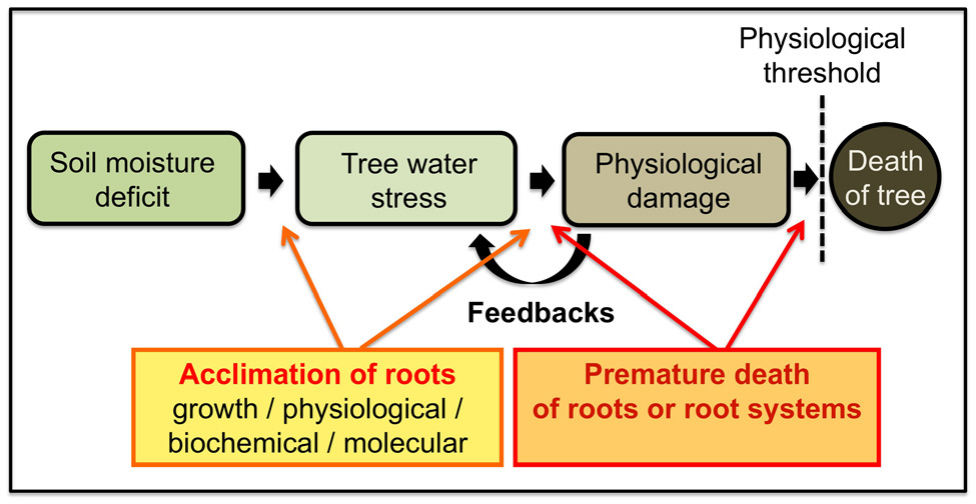
**Schematic diagram illustrating how soil moisture deficit results in physiological damage and ultimately in tree death.** The balance between growth and survival is tightly regulated, and specific acclimation mechanisms have evolved to allow growth under drought conditions. When the intensity of drought increases, steady state conditions of water transfer may be irreversibly disrupted, resulting in hydraulic failure and, thus, in premature death of roots or root systems (based on Waring, 1987; Breda et al., 2006; Anderegg et al., 2013a; Claeys and Inzé, 2013).

As the intensity of drought increases, steady state conditions of water transfer in the xylem tissues may be irreversibly disrupted, due to water cohesion breakdown and massive xylem embolism (Anderegg et al., 2013a). The water stress of trees is then apparent as physiological damage that is associated with a number of characteristics, processes, and feedbacks that can impact tree health at various time scales (**Figure 3**). One feedback of hydraulic failure is premature mortality of roots or root systems (Waring, 1987; Breda et al., 2006), which leaves a tree more vulnerable to drought the following year. In particular, damage that limits a tree’s ability to make use of water or nutrients when they briefly become available again could interact with multi-year drought or other stressors to cause system failure (Anderegg et al., 2013b). Further, if a tree accumulates enough physiological damage, particularly over several years, and if the physiological damage crosses some threshold, tree death may occur (Anderegg et al., 2013b).

Increased mortality of trees during and after drought has been observed in recent years (van Mantgem et al., 2009; Anderegg et al., 2013b). However, the mortality process in trees is poorly understood, as indicated by Meir et al. (2015), and the question of how exactly trees are killed by drought remains unanswered (Hartmann et al., 2013a). Drought affects both tree hydraulics and C balance because trees, as with all vascular plants, respond to decreasing soil water availability with stomatal closure, thereby reducing C assimilation rates. Consequently, long-lived plants such as trees might be forced into a negative C balance, by mobilizing stored C to fulfill metabolic needs, until reserves are eventually depleted (McDowell et al., 2008; Sala et al., 2010). The physiological response of C depletion is closely linked to C reserves, particularly in the root system, which is a large sink for NSC. Roots are entirely dependent on the autotrophic parts of the plant and might require up to half of all the photosynthates produced (Lambers et al., 2008). A drought-stress study of the two poplar species *Populus tremuloides* and *P. balsamifera* indicated that reduced reserve accumulation in the root system during drought decreases the conversion of starch to SSs in roots (Galvez et al., 2013). This response probably contributes to the root death of drought-exposed poplars during the dormant season by compromising the frost tolerance of the root system.

However, recent evidence indicates that, under water deficit, the growth of sink organs such as roots is uncoupled from photosynthesis (Muller et al., 2011). There is usually a robust correlation between C availability and the growth of sink organs, but this relationship becomes weaker or is modified under drought conditions (Muller et al., 2011). Moreover, drought kills trees much faster than C starvation, and pools of stored C, in roots as well as in other organs, are not depleted at the time of death for drought-stressed trees the way they are for C starved, well-watered trees (Hartmann et al., 2013a). Investigating additional mechanisms of tree death due to drought, e.g., pathogen attack could be a priority in future research as well.

Root mortality, as mentioned above, is not necessarily linked to the mortality of the whole organism. In particular, some specific woody perennials have evolved strategies that enable them to overcome extreme stresses including drought and to become essentially immortal, e.g., by vegetative reproduction as in the proteaceous shrub *Lomatia tasmanica* (Munné-Bosch, 2014).

## Conclusion

As one of the major disturbances in forest ecosystems, drought forces tree roots to react within a short period to this changed condition with growth, physiological, biochemical, and molecular responses. Reduced fine root biomass is one of the most common growth/morphological responses in trees. Other root traits, such as SRL, RTD, or the C/N ratio, are only slightly affected or remain unchanged. This indicates that tree roots have evolved effective strategies to coordinate the complex metabolic and structural demands required to acclimate and to maintain physiological and morphological functions when faced with drought conditions. However, trees experiencing drought invest large amounts of C in defense and storage systems, such as lignified support tissues, rather than in attaining external resources. This increased allocation to mechanisms for tolerating damage and disturbance can impact the competitive ability of a tree (see also Dietze et al., 2014).

Future research concerning tree roots and drought should focus on establishing accurate and commonly accepted approaches for understanding root functions. Although we are able to measure fine root biomass after destructive sampling, it is still not possible to estimate this parameter without physical damage. Other parameters, such as fine root lifespan and turnover rate, are even more difficult to measure, and the options of indirect quantification via sequential coring, direct measurement using minirhizotrons, and (the most recent method) radiocarbon (^14^C bomb fallout) techniques all have important restrictions (for reviews see Gaudinski et al., 2010; Lukac, 2012; Ahrens et al., 2014; McCormack and Guo, 2014). More research attention should also be given to the biochemical characterization of tree fine roots. To our knowledge, the lignin and suberin situation in living fine roots of trees after drought has only rarely, if ever, been studied. Does rebuilding of root structure occur only after a severe drought or mainly after repeatedly occurring droughts? How fast is this process? These root traits could be monitored over time using biomarkers, yet we are not aware of any existing applications of this relatively simple approach. Lignification or suberisation of tree roots has an obvious long-term effect on the decomposability of roots, and hence on the quality of SOM. Overall, a thorough understanding of the terrestrial biosphere and C cycle under the changing climate clearly requires more research on the ‘hidden half’ that exists below ground.

## Conflict of Interest Statement

The authors declare that the research was conducted in the absence of any commercial or financial relationships that could be construed as a potential conflict of interest.
